# Role of insulin-regulated aminopeptidase as potential biomarker in insulin resistant polycystic ovary syndrome patients

**DOI:** 10.20945/2359-4292-2026-0011

**Published:** 2026-02-16

**Authors:** Osman Köse, Koray Gök, Elif Köse, Sezen Irmak Gözükara, Abdullah Tüten, Mehmet Sühha Bostancı

**Affiliations:** 1 Department of Obstetrics and Gynecology, Sakarya University Faculty of Medicine, Sakarya, Turkey; 2 Department of Obstetrics and Gynecology, Cerrahpasa Faculty of Medicine, Istanbul University-Cerrahpasa, Istambul, Turkey; 3 Department of Public Health, Sakarya University Faculty of Medicine, Adapazarı, Sakarya, Turkey; 4 Clinic of Medical Biochemistry, Sakarya Training and Research Hospital, Adapazarı, Sakarya, Turkey

**Keywords:** Polycystic ovary syndrome, Insulin, Insulin resistance

## Abstract

**Objective:**

To measure serum insulin-regulated aminopeptidase levels in women diagnosed with polycystic ovary syndrome and to investigate their potential contribution of these levels to the development of insulin resistance, which plays a central role in the pathophysiology of polycystic ovary syndrome.

**Methods:**

The study group, recruited between May and December 2021, consisted of 40 patients diagnosed with polycystic ovary syndrome and 40 age-matched healthy controls. Serum insulin-regulated aminopeptidase levels were compared between the groups using the ELISA method.

**Results:**

Serum insulin-regulated aminopeptidase levels were significantly lower in the polycystic ovary syndrome group compared with the control group (p < 0.001). Subparameter assessments revealed that insulin-regulated aminopeptidase levels were even lower in insulin-resistant polycystic ovary syndrome patients (p = 0.001). Moreover, insulin-regulated aminopeptidase levels demonstrated a statistically significant negative correlation with fasting blood glucose, insulin, glycated hemoglobin, and HOMA-IR values.

**Conclusion:**

Serum insulin-regulated aminopeptidase levels were found to be lower in women with polycystic ovary syndrome than those in healthy controls. Furthermore, these levels appear to reflect insulin resistance, a key factor in the pathogenesis of polycystic ovary syndrome. Overall, these findings suggest that insulin-regulated aminopeptidase may serve as a potential biomarker for the identifification of insulin resistance in women with polycystic ovary syndrome.

## INTRODUCTION

Polycystic ovary syndrome (PCOS) is one of the most common endocrine disorder among women of reproductive age, affecting approximately 11 to 13% of the global female population ^(1,2)^. It is a heterogeneous condition characterized by three core diagnostic features – hyperandrogenism, chronic anovulation, and polycystic ovarian morphology – often accompanied by metabolic abnormalities such as insulin resistance (IR) and obesity ^(3)^. Although the pathophysiology of PCOS is not fullyunderstood, it is thought to involve a combination of genetic predisposition, environmental factors, oxidative stress, chronic low-grade inflammation, and metabolic dysregulation ^(3,4)^. Insulin resistance, which plays a central role in the etiology of the disease, is present in approximately 50 to 80% of patients and contributes to hyperandrogenemia and reproductive dysfunction through multiple mechanisms ^(5,6)^. However, the exact mechanisms underlying IR in PCOS remain unclear, warranting further research ^(4)^. It has long been established that alterations in glucose transporters (GLUT) are involved in the development of IR. The primary mechanism is insulin-stimulated cellular glucose uptake, which is facilitated by the translocation of GLUT4-containing vesicles from the cytoplasm to the plasma membrane ^(7)^. Insulin-regulated aminopeptidase (IRAP), a cellular protein featuring both intracellular and extracellular functional sites, is essential for this translocation process and plays a critical role in insulin-mediated glucose uptake via GLUT-4 in skeletal muscle and adipose tissue. During this process, its extracellular domain is cleaved by metalloproteases and released into the bloodstream ^(8)^. Given these functions, circulating IRAP levels have been proposed as a potential biomarker for IR, as demonstrated in previous studies in patients with type 2 diabetes ^(8,9)^. However, a review of the existing literature indicates that this association has not yet been investigated in women with PCOS.

For these reasons, this study aimed to measure serum IRAP levels in women diagnosed with PCOS and to investigate their potential contribution of these levels to the development of IR, which plays a central role in the pathophysiology of PCOS.

## METHODS

This case-control study was conducted between May and December 2021 at the Department of Obstetrics and Research Hospital. Ethical approval was obtained from the Ethics Committee of the Faculty of Medicine, Sakarya University (date: May 7, 2021, approval no: 90). The study was conducted in accordance with the principles of the Declaration of Helsinki, and written informed consent was obtained from all volunteers.

The study included 40 patients diagnosed with PCOS according to the Rotterdam criteria (2003). The control group consisted of 40 healthy women of similar age and body mass index (BMI) who presented to the clinic for routine gynecological examinations. The inclusion criteria for all participants were an age range of 18 to 40 years and a BMI between 20 to 35 kg/m^2^. For the control group, the absence of any significant gynecological pathology was required; only minor complaints such as dysmenorrhea or premenstrual syndrome were permitted.

The exclusion criteria for all participants were as follows: a history of smoking; current pregnancy or lactation; previous ovarian surgery; a diagnosis of endometrioma or endometriosis; thyroid disorders or abnormal prolactin levels; use of hormonal therapy (e.g., oral contraceptives) within the past 6 months; use of medications known to affect carbohydrate metabolism (e.g., insulin, cortisol); a history of chronic disease (e.g., epilepsy, renal failure, heart disease); and a history of cancer.

Clinical data and laboratory results of the participants were retrospectively collected from medical records. The following parameters were recorded: age, height, weight, intermenstrual interval, and the presence of oligo-anovulation and hirsutism. Body mass index was calculated using the formula: BMI = weight (kg) / [height (m)]^2^.

The diagnosis of PCOS was established according to the Rotterdam criteria, which requires the presence of at least two of the following three features:

- Oligo-ovulation or amenorrhea, defined as a menstrual cycle exceeding 35 days or the absence of menses for 3 consecutive months, respectively.
- Clinical hyperandrogenism, primarily evidenced by hirsutism. This was assessed using the modified Ferriman-Gallwey (mFG) scoring system, where nine androgen-sensitive body areas were assigned a score from zero (no terminal hair growth) to 4 (extensive hair growth). A total mFG score of ≥ 8 was considered diagnostic ^(11)^.
- Biochemical hyperandrogenism, defined as an elevated serum level of any of the following androgens: total testosterone (reference range: 0.04 to 4.18 ng/dL), dehydroepiandrosterone sulfate (DHEAS) (reference range: 10 to 248 µg/dL), or 17-OH-progesterone (reference range: 0.2 to 1.0 ng/mL) ^(12-14)^. All participants underwent routine gynecological and transvaginal ultrasonographic examinations during the early follicular phase (days 2 to 4) of a spontaneous menstrual cycle. For the diagnosis of PCOS, polycystic ovarian morphology was defined according to the Rotterdam criteria as the presence of ≥ 20 follicles (2 to 9 mm in diameter) per ovary and/or an ovarian volume > 10 mL ^(15)^. The control group consisted of healthy women with regular menstrual cycles (26 to 32 days), normal ovarian morphology, and no evidence of clinical or biochemical hyperandrogenism.

### Laboratory studies

Blood samples were obtained from the brachial vein during the early follicular phase (cycle days 2 to 4) following an 8-hour overnight fast. A total of 16 mL of venous blood was collected from each participant into two tubes in the early morning. From one tube, the following serum analyses were performed: anti-Müllerian hormone (AMH), follicle-stimulating hormone (FSH), luteinizing hormone (LH), thyroid-stimulating hormone (TSH), total testosterone, free testosterone, DHEAS, 17-hydroxyprogesterone (17-OHP), fasting glucose, and fasting insulin. Serum AMH levels were quantified using a chemiluminescent immunoassay (Access AMH kits, Beckman Coulter Access 2 analyzer). This assay had a measurement range of 0.02 to 24 ng/mL, with an intra-assay coefficient of variation (CV) of 1.7% and an inter-assay CV of 3.1%. FSH, LH, TSH, total testosterone, insulin, and DHEAS levels were measured by chemiluminescent immunoassay on an Abbott Architect i2000 analyzer (Abbott Diagnostics). The inter-assay CVs were as follows: ≤ 10% for FSH, ≤ 7% for LH, ≤ 10% for TSH, ≤ 10% for total testosterone, ≤ 7% for insulin, and ≤ 10% for DHEAS. Fasting glucose levels were determined via the hexokinase method on a Beckman Coulter AU5800 analyzer. The assay demonstrated an intra-assay CV of 0.7%, a total CV of 0.9%, and a measurement range of 10 to 800 mg/dL. Serum 17-OHP and free testosterone levels were measured using radioimmunoassays (RIA) with Diasource kits. For the 17-OHP assay, the intra-assay CV was 6.8%, the inter-assay CV was 8.7%, and the measurement range was 0.17 to 14 ng/mL. For the free testosterone assay, the intra-assay CV was 5.7%, the inter-assay CV was 7.3%, and the measurement range was 0.3 to 90 pg/mL. Insulin resistance was evaluated using the homeostasis model assessment of insulin resistance (HOMA-IR), calculated as [fasting insulin (µU/mL) × fasting glucose (mg/dL)] / 405. A HOMA-IR value ≥ 2.5 was defined as indicative of IR.

The blood samples taken with the second tube blood sample were centrifuged at 4,000 rpm for 10 minutes, then the serums were separated and put into a deep freezer to be stored at -80°C until the day of analysis. On the study day, all samples were thawed in the same month and Human ICE protease-activating factor (IRAP) levels were measured using ELISA kits (MyBioSource, San Diego, USA, Catalog no: MBS260922). The results were calculated using the Biotek ELX800 (USA) ELISA reader, while the intra-measurement CV was reported as < 8% and the inter-measurement coefficient of variation as < 12%.

### Statistics

Descriptive statistics are presented as mean ± standard deviation for normally distributed continuous variables and median (first-third quartiles) for non-normally distributed variables. Comparisons between groups for parametric and non-parametric data were performed using the Student’s *t*-test and the Mann-Whitney U test, respectively. Correlations between continuous variables were assessed using Pearson’s correlation coefficient. The discriminatory power of IRAP for diagnosing PCOS was evaluated using Receiver Operating Characteristic (ROC) curve analysis. A p-value of less than 0.05 was considered statistically significant. All analyses were conducted using IBM Statitical Package for the Social Sciences (SPSS) statistics, Version 20.0.

## RESULTS

The clinical and laboratory findings of the participants in the patient and control groups included in this study are shown in **Table 1**. Serum LH, total testosterone, AMH, DHEAS, fasting glucose, insulin, and HOMA-IR values were significantly higher in the PCOS group than the control group. In contrast, serum IRAP levels were significantly lower in the PCOS group compared to the control group. Laboratory data comparing PCOS patients with and without IR are shown in **Table 2**. Accordingly, serum IRAP levels were significantly lower in the insulin-resistant group. The correlation of serum IRAP levels with biochemical variables is presented in **Table 3**. IRAP levels show a significant negative correlation with insulin, HbA1c, fasting blood glucose levels, and HOMA-IR.

**Table 1.** Comparison of the clinical and laboratory characteristics of the women with polycystic ovary syndrome and the control group

	PCOS Median (1.-3. Quarter)	Control Median (1.-3. Quarter)	p-value
Age, years	27,00 (25,00-29,00)	28,00 (24,00-30,00)	0.674*
BMI, kg/m	26,82 ± 4,08	25,00 ± 4,27	0.054^†^
Serum fasting glucose, mg/dL	95,98 ± 7,80	89,90 ± 7,98	0.001^†^
LH, mIU/mL	11,47 ± 3,94	6,98 ± 2,62	< 0.001^†^
AMH, ng/mL	10,11 (6,87-13,48)	7,22 (5,11-8,54)	< 0.001*
FSH, mIU/mL	6,03 (5,19-7,31)	5,67 (5,00-6,00)	0.023*
TSH, uIU/mL	1,76 (1,19-2,46)	1,78 (1,42-2,49)	0.893*
Prolactin, ng/mL	14,80 (10,33-19,50)	13,15 (9,71-18,43)	0.587*
HDL, mg/dL	56,00 (48,36-63,25)	51,22 (43,25-61,25)	0.178*
LDL, mg/dL	118,50 (100,25-145,00)	105,00 (98,00-121,50)	0.204*
Cholesterol, mg/dL	157,80 ± 28,98	154,18 ± 17,85	0.504^†^
Triglycerides, mg/dL	86,00 (66,50-119,75)	69,07 (54,25-95,00)	0.030*
17-OH Progesterone, ng/mL	1,28 (0-87-2,02)	0,70 (0,57-0,87)	< 0.001*
Free testosterone, pg/mL	2,20 (1,74-3,01)	1,84 (1,38-2,17)	0.001*
Total testosterone, ng/dL	35,75 (28,13-48,68)	24,95 (20,57-31,57)	< 0.001*
DHEAS, µg/dL	318,81 ± 90,08	233,19 ± 66,05	< 0.001^†^
F-G score	13,00 (11,00-15,75)	7,00 (6,00-8,00)	< 0.001*
HOMA-IR	3,45 ± 1,46	1,60 ± 0,93	< 0.001^†^
Insulin, µIU/mL	13,90 ± 4,94	7,19 ± 4,00	< 0.001*
HbA1c	5,83 ± 0,43	5,16 ± 0,35	< 0.001^†^
IRAP, ng/mL	0,47 ± 0,19	0,73 ± 0,17	< 0.001^†^

* Mann-Whitney U Test,

† Student t-test.

Results expressed as mean ± standard deviation.

POCUS: polycystic ovary syndrome; BMI: body mass index; LH: luteinizing hormone; AMH: Anti-Müllerian hormone; FSH: follicle-stimulating hormone; TSH: thyroid-stimulating hormone; HDL: high-density lipoprotein; LDL: low-density lipoprotein; DHEAS: dehydroepiandrosterone sulfate; F-G score: Ferriman-Gallwey score; HOMA-IR: homeostatic model assessment for insulin resistance; HbA1c: glycated hemoglobin; IRAP: insulin-regulated aminopeptidase.

**Table 2.** Comparison of laboratory tests in insulin resistant and non-insulin resistant polycystic ovary syndrome patients according to the homeostatic model assessment for insulin resistance cut-off of 2.5

	PCOS Insulin resistance +	PCOS Insulin resistance -	p-value*
Serum fasting glucose, mg/dL	104,04 ± 6,01	90,21 ± 4,55	< 0.001
Serum fasting insulin, µIU/mL	17,09 ± 3,66	9,11 ± 1,50	< 0.001
HOMA-IR	4,41± 1,09	2,03 ± 0,34	< 0.001
HbA1c, %	6,11 ± 0,23	5,42 ± 0,32	< 0.001
IRAP, ng/mL	0,39 ± 0,16	0,59 ± 0,16	0.001

* Student t test.

POCUS: polycystic ovary syndrome; HOMA-IR: homeostatic model assessment for insulin resistance; HbA1c: glycated hemoglobin; IRAP: insulin-regulated aminopeptidase.

**Table 3.** Correlation of serum insulin-regulated aminopeptidase levels with biochemical variables in the polycystic ovary syndrome group

PCOS-IRAP	r	p-value
Serum fasting glucose, mg/dL	-0.604	< 0.001
HOMA-IR	-0.642	< 0.001
Insulin, µIU/mL	-0.617	< 0.001
HbA1c, %	-0.507	0.001
Free testosterone, pg/mL	-0.221	0.170
Total testosterone, ng/dL	-0.276	0.085
SDHEA, µg/dL	0.194	0.231

r: Pearson correlation coefficient.

HOMA-IR: homeostatic model assessment for insulin resistance; HbA1c: glycated hemoglobin; SDHEA: serum dehydroepiandrosterone sulfate.

**Figure 1:**
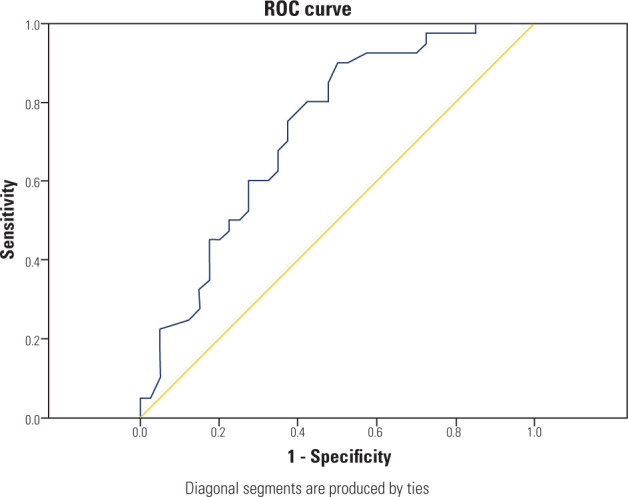
Receiver Operating Characteristic analysis of the discrimination of insulin-regulated aminopeptidase values of polycystic ovary syndrome cases.

The ROC curve for IRAP concentrations in the PCOS group is shown in **Figure 1**. The area under the curve was 83.1% (95% confidence interval: 0.744-0.918). The optimal cut-off value was 0.655 ng/mL, and the rates below this value were 82.5% and 70.0% for sensitivity and specificity, respectively (p=0.001).

ROC: Receiver Operating Characteristic.

## DISCUSSION

Our results demonstrate that serum IRAP levels are significantly lower in the PCOS group than in the control group. Notably, a further reduction in IRAP levels was observed in PCOS patients with IR relative to those without, suggesting a significant influence of IR on IRAP expression. These findings suggest that IRAP may be associated not only with IR but also with hormonal disorders. Significant negative correlations were also observed between serum IRAP levels and fasting insulin, fasting glucose, HbA1c, and HOMA-IR. These correlations suggest that decreased IRAP levels are linked to IR and dysregulated glucose metabolism, positioning IRAP as a potential biomarker for IR. This finding aligns with existing literature. Furthermore, weak negative correlations were identified between IRAP levels and serum DHEAS, free testosterone, and total testosterone concentrations in PCOS patients. These findings suggest that IRAP may be associated not only with IR but also with hormonal disorders. The interactions between insulin, GLUT-4, and its closely associated protein, IRAP, are integral to regulating cellular glucose uptake, maintaining whole-body glucose homeostasis, and ensuring metabolic equilibrium. Disruptions in this intricate process can lead to IR. A review of the literature indicates that previous investigations into GLUT-4 and its role in IR in PCOS patients have relied on invasive methods, such as biopsies of adipocyte and endometrial tissue ^(16-18)^. As a less invasive alternative to these approaches, we hypothesized that measuring serum IRAP levels could serve as a surrogate marker for GLUT-4 translocation to the plasma membrane and, by extension, provide a reflection of IR in PCOS patients. This premise formed the rationale for the present study.

A review of the literature indicates that numerous studies have investigated the relationship between IR and IRAP. Research has demonstrated reduced IRAP translocation in both diabetic rats 19 and patients with type 2 diabetes mellitus (T2DM) ^(20,21)^. These foundational studies, which utilized invasive biopsy methods to analyze adipocyte ^(19,21)^ and skeletal muscle tissue ^(20)^, established a clear link between impaired IRAP activity and IR. Subsequent clinical investigations have employed a more practical, non-invasive approach by measuring serum IRAP levels. These studies consistently report decreased circulating IRAP levels in individuals with T2DM ^(8,9)^ and in pregnant women with gestational diabetes ^(22)^. Collectively, these findings from both tissue-based and serum-based analyses suggest that reduced IRAP levels may serve as a useful indicator of IR.

In our study, we found that the serum IRAP levels were significantly lower in the insulin-resistant group. It is well-established that insulin stimulation triggers the translocation of IRAP and GLUT4 to the plasma membrane as a complex. Subsequently, the extracellular domain of IRAP is cleaved by metalloproteases and released into the circulation ^(8,23)^. In states of IR, this physiological process is disrupted, leading to reduced cleavage and secretion of IRAP and, consequently, lower circulating levels. The findings of the present study are consistent with this mechanism and align with previous reports in the literature.

The PCOS group exhibited significantly higher levels of IR markers, including fasting insulin, fasting glucose, HbA1c, and HOMA-IR, relative to healthy controls. Consistent with this metabolic profile, serum IRAP levels showed a significant negative correlation with these indices. This inverse association provides important evidence linking lower IRAP levels to the development of IR in PCOS.

As a result of our study, we found that serum IRAP levels were significantly lower in the insulin-resistant group. Moreover, IRAP levels demonstrated strong and statistically significant negative correlations with metabolic parameters, including fasting glucose, insulin, HOMA-IR, and HbA1c, indicating that higher IR and poorer glycemic control are associated with lower IRAP levels in PCOS patients. In contrast, IRAP levels showed weak and statistically non-significant correlations with hormonal parameters, including free and total testosterone and DHEA-S. Overall, these findings suggest that IRAP is more closely associated with metabolic disturbances than with androgen levels in PCOS, implying that circulating IRAP levels may primarily reflect IR, while their potential relationship with other hormonal parameters warrants further investigation in future studies.

The main limitations of this study include its case-control design, the relatively small sample size, the limited number of participants in the PCOS subgroups, and the use of the HOMA-IR index rather than the hyperinsulinemic-euglycemic clamp, which is considered the gold-standard method for assessing IR. An additional limitation is that the ACTH stimulation test was not performed to definitively rule out non-classical congenital adrenal hyperplasia (in patients with elevated 17-OH progesterone levels. Although clinical evaluation and DHEAS levels did not indicate non-classical congenital adrenal hyperplasia, future studies should incorporate this test to provide a more definitive differential diagnosis.

## CONCLUSION

This study provides the first evidence linking reduced serum insulin-regulated aminopeptidase levels to polycystic ovary syndrome. Notably, insulin-regulated aminopeptidase levels were significantly lower in polycystic ovary syndrome patients both with and without insulin resistance compared to controls. The significant negative correlations observed between insulin-regulated aminopeptidase levels and indices of both insulin resistance and hyperandrogenemia indicate that insulin-regulated aminopeptidase may be involved in the core metabolic and endocrine disturbances characterizing the pathogenesis of polycystic ovary syndrome.

## Data Availability

datasets related to this article will be available upon request to the corresponding author.
