# Erratum: Role of insulin-regulated aminopeptidase as potential biomarker in insulin resistant polycystic ovary syndrome patients

**DOI:** 10.20945/2359-4292-2026-0071

**Published:** 2026-07-03

In the article **“Role of insulin-regulated aminopeptidase as potential biomarker in insulin resistant polycystic ovary syndrome patients”**, published in Archives of Endocrinology and Metabolism, DOI: 10.20945/2359-4292-2026-0011:

Page 3, subtitle: Laboratory studies

Where it reads:

The blood samples taken with the second tube blood sample were centrifuged at 4,000 rpm for 10 minutes, then the serums were separated and put into a deep freezer to be stored at -80°C until the day of analysis. On the study day, all samples were thawed in the same month and Human ICE protease-activating factor (IRAP) levels were measured using ELISA kits (MyBioSource, San Diego, USA, Catalog no: MBS260922). The results were calculated using the Biotek ELX800 (USA) ELISA reader, while the intra-measurement CV was reported as < 8% and the inter-measurement coefficient of variation as < 12%.

Read:

**The blood samples taken with the second tube blood sample were centrifuged at 4,000 rpm for 10 minutes, then the serums were separated and put into a deep freezer to be stored at -80°C until the day of analysis. On the study day, all samples were thawed simultaneously and Human Leucyl-cystinyl Aminopeptidase (LNPEP) levels were measured using ELISA kits (BT LAB, Zhejiang, China; Catalog no: E74293Hu). Absorbance values were measured at 450 nm using a Biotek ELX800 microplate reader (BioTek Instruments, USA), and concentrations were calculated from the standard curve according to the manufacturer’s instructions. The intra-assay coefficient of variation was <8% and the inter-assay coefficient of variation was <10%, as reported by the manufacturer.**

The correction refers exclusively to the description of the assay kit used in the Methods section and does not affect the results, analyses, or conclusions of the study.

**Editor in chief:**

César Boguszewski

https://orcid.org/0000-0001-7285-7941

